# Implementation of the Montessori model as a non‐pharmacological intervention for dementia in Latin America

**DOI:** 10.1002/alz70858_106188

**Published:** 2025-12-25

**Authors:** Jorge M Leon‐Salas

**Affiliations:** ^1^ Clínica Bíblica Hospital, San José, Costa Rica; Global Brain Health Institute, Trinity College Dublin, dublin, Ireland

## Abstract

Almost 9.9 million people develop dementia each year, the majority (63%) of whom reside in low‐ and middle–income countries. In Latin America, this represents a major burden for health systems and society, that directly impacts quality of life of those living with dementia, family members and caregivers. Therefore, in the absence of accessible pharmacological strategies for neurodegenerative disease, a renewed interest has emerged in non‐pharmacological interventions, which enhance a more dignifying way of living. This encourages a shift towards solutions that are local and community‐based, without requiring complex interventions of highly trained personnel.

After many years of accumulated knowledge in the Montessori Model of Learning, researchers have adapted basic principles for people living with dementia. As a person‐centered initiative, the intervention aims to promote independence, higher self‐esteem, self‐agency and an active involvement in society, while taking into account the unmet needs of neurodegeneration. This philosophy fosters a constructive and rewarding environment, which is especially valuable in long term care facilities and cases where patients may be experiencing reactive behaviors.

We will provide an overview of the Montessori Approach for Dementia, focusing on easily implemented strategies and techniques.

